# Predictive ability of the Desire to Avoid Pregnancy scale

**DOI:** 10.1186/s12978-023-01687-9

**Published:** 2023-09-25

**Authors:** Jennifer A. Hall, Geraldine Barrett, Judith Stephenson, Corinne H. Rocca, Natalie Edelman

**Affiliations:** 1 Reproductive Health Research Department, UCL Elizabeth Garrett Anderson Institute for Women’s Health, London, UK; 2 San Francisco (UCSF) School of Medicine, Department of Obstetrics, Gynecology and Reproductive Sciences, Advancing New Standards in Reproductive Health (ANSIRH), University of California, San Francisco, USA; 3 Independent Researcher and Trauma-Informed Consultant at TRuST, Brighton, UK

## Abstract

**Background:**

A longstanding gap in the reproductive health field has been the availability of a screening instrument that can reliably predict a person’s likelihood of becoming pregnant. The Desire to Avoid Pregnancy Scale is a new measure; understanding its sensitivity and specificity as a screening tool for pregnancy as well as its predictive ability and how this varies by socio-demographic factors is important to inform its implementation.

**Methods:**

This analysis was conducted on a cohort of 994 non-pregnant participants recruited in October 2018 and followed up for one year. The cohort was recruited using social media as well as advertisements in a university, school, abortion clinic and outreach sexual health service. Almost 90% of eligible participants completed follow-up at 12 months; those lost to follow-up were not significantly different on key socio-demographic factors. We used baseline DAP score and a binary variable of whether participants experienced pregnancy during the study to assess the sensitivity, specificity, area under the ROC curve (AUROC) and positive and negative predictive values (PPV and NPV) of the DAP at a range of cut-points. We also examined how the predictive ability of the DAP varied according to socio-demographic factors and by the time frame considered (e.g., pregnancy within 3, 6, 9 and 12 months).

**Results:**

At a cut-point of 2 on the 0–4 range of the DAP scale, the DAP had a sensitivity of 0.78, a specificity of 0.81 and an excellent AUROC of 0.87. In this sample the cumulative incidence of pregnancy was 16% (95%CI 13%, 18%) making the PPV 43% and the NPV 95% at this cut-point. The DAP score was the factor most strongly associated with pregnancy, even after age and number of children were taken into account. The association between baseline DAP score and pregnancy did not differ across time frames.

**Conclusions:**

This is the first study to assess the DAP scale as a screening tool and shows that its predictive ability is superior to the limited pre-existing pregnancy prediction tools. Based on our findings, the DAP could be used with a cut-point selected according to the purpose.

**Supplementary Information:**

The online version contains supplementary material available at 10.1186/s12978-023-01687-9.

## Introduction

A longstanding gap in the reproductive health field has been the availability of a screening instrument that can reliably predict a person’s likelihood of becoming pregnant. Such an instrument would be of particular use for researchers either trying to identify a specific cohort (e.g. for preconception research) or conducting research for which it is important to exclude participants who are likely to become pregnant, (e.g. some pharmacological studies). The Desire to Avoid Pregnancy (DAP) scale is a psychometric instrument that measures a person’s preferences about a potential future pregnancy. The DAP was developed as a research instrument in the USA [1] and was validated for use in the UK in 2022 [2]. This 14-item tool has been shown to be associated with contraceptive use in two US studies [3, 4] and was shown to have good predictive ability for pregnancy occurring within one year in the UK. Women with the lowest desire to avoid pregnancy (a score of zero) had an 80% chance of becoming pregnant within 12 months compared to < 1% in women with the highest desire to avoid pregnancy (a score of four). To our knowledge the DAP scale is the only purposively designed and evaluated prospective measure of pregnancy preferences.

The DAP is a new measure and understanding its potential use as a screening tool and ability to predict pregnancy is important for both research and clinical purposes. While the overall predictive ability over 12 months appears to be high, further exploration is required to understand its sensitivity and specificity relative to incident pregnancy, and whether the predictive ability of the DAP varies according to sociodemographic factors, as well as over different timeframes [5–7].

The aims of this paper are to: (1) to examine the sensitivity and specificity of the DAP scale relative to incident pregnancy; and (2) to explore the scale’s predictive ability across socio-demographic characteristics and time frames.

## Methods

### Sample

This analysis was conducted on a cohort of 994 non-pregnant participants recruited in October 2018 and followed up for one year. The full details of recruitment and participation are described elsewhere [2] but, in brief, people who self-reported as female, were pre-menopausal and not sterilised, aged 15 years or over and living in the United Kingdom, were recruited though a mixture of site-based advertising (school, university, sexual health and pregnancy termination clinics) and online recruitment through both paid advertisements (Instagram and Facebook) and sharing through researchers’ and participants’ networks. Participants completed an online RedCap survey at baseline and every three months for 12 months [8, 9] that included the DAP scale and other questions about pregnancy preferences, contraceptive use, preconception preparation and socio-demographics. At each of the three-month follow-up surveys, participants were asked whether they were currently pregnant or had been pregnant since the last survey.

### Measures

#### Outcome

Our outcome was experience of an incident pregnancy over 12 months (yes/no), created using self-reported pregnancy data across all follow-up surveys. For analyses of time frame, we also looked at incident pregnancy by 3, 6, and 9 months, individually.

#### DAP scale

The DAP scale is a psychometrically validated measure of a woman’s preferences about a potential future pregnancy, developed using an extensive item development process and item response theory to create the final tool [1]. Its 14 items cover three conceptual domains: (1) cognitive desires and preferences; (2) affective feelings and attitudes; and (3) anticipated practical consequences. Each of the 14 items asks respondents to report using a Likert scale on how much they agree or disagree with a statement about either becoming pregnant in the next three months or having a baby in the next year (see Additional file 1 for the complete wording of the DAP). Each item is scored zero to four; responses are summed and averaged to get a total score between zero and four, with four representing the greatest desire to avoid pregnancy and zero the most open to pregnancy.

### Analysis

#### Sensitivity and specificity of the DAP relative to pregnancy

The relative importance of sensitivity and specificity vary according to the purpose of using the DAP scale (i.e. whether identifying who will become pregnant (sensitivity) is more important than identifying who will not (specificity) or vice versa), therefore a range of cut-points was explored. Initially the Youden index was used to suggest an empirically optimal cut-point, i.e. the best balance of sensitivity and specificity [10]. This cut point was used to classify women as ‘test positive’ if their score was below the cut-point and was compared with the ‘true positive’ of whether they experienced a pregnancy between baseline and 12months. The sensitivity, specificity, area under the receiver operator curve (AUROC) and positive and negative predictive values (PPV and NPV) were then calculated. The AUROC represents the DAP’s ability to discriminate between those who will and will not become pregnant, where 0.5 is no better than random and 1.0 is perfect discrimination. An AUROC of 0.7–0.8 was considered acceptable, 0.8–0.9 excellent and > 0.9 outstanding [11]. This process was then repeated for a range of cut-points to provide information to enable the selection of the most suitable cut-point depending on purpose.

#### Predictive ability of the DAP

##### Univariate analysis

Univariate analyses were conducted to explore how pregnancy preferences, as measured by DAP score, vary by age, ethnicity, education, number of children and relationship status, using the Kruskall-Wallis test for ordered categorical variables (where DAP score was not expected to increase or decrease consistently (age group, number of children in the household, ethnicity)) and the Kendall’s tau where it was (education, relationship status). Non-parametric tests were used given the non-normal distribution of the DAP score. Baseline data were used for all socio-demographic factors as there was minimal change over follow up. The relationship between each factor and occurrence of pregnancy was also examined with logistic regression. We examined differential attrition by socio-demographics and baseline DAP score using t-tests, Kruskall-Wallis and chi-squared tests, as appropriate.

##### Multivariable analysis

A multivariable logistic regression model of DAP score as a predictor of pregnancy was created by including all the socio-demographic factors considered and removing them in a manual backwards stepwise process, starting with the largest p-value and retaining only variables where p < 0.1. The predicted probabilities of pregnancy from this model were examined, both overall and by age and number of children.

#### Timeframes

To determine whether the predictive ability of DAP score varies by the time frame considered, and therefore whether asking preferences annually is sufficient or should be done more frequently, the baseline DAP score was used to calculate the odds pregnancy of between baseline and three, six, nine and twelve months respectively using logistic regression. Given low attrition and to ease interpretation, we include participants in pregnancy denominators until they were lost from the cohort and report percentages rather than rates.

## Results

### Sample

As described previously [2], the baseline cohort of 994 women were aged 15–50 years (median 31, IQR 23–36, mean 29.7). Most were white (84%), described themselves as heterosexual (82%) and were in a relationship (82%). Over half (57%) had one or more children in the household, 25% had completed secondary school, 39% had an undergraduate degree and 31% had postgraduate or other professional qualifications.

Almost 90% (831/929) of participants eligible to take part in the 12month survey did so. The women who did not take part in the 12month follow up survey did not differ by age, ethnicity, relationship status, number of children or baseline DAP score (see Additional file 2).

### Sensitivity, specificity of the DAP

**Fig. 1:**
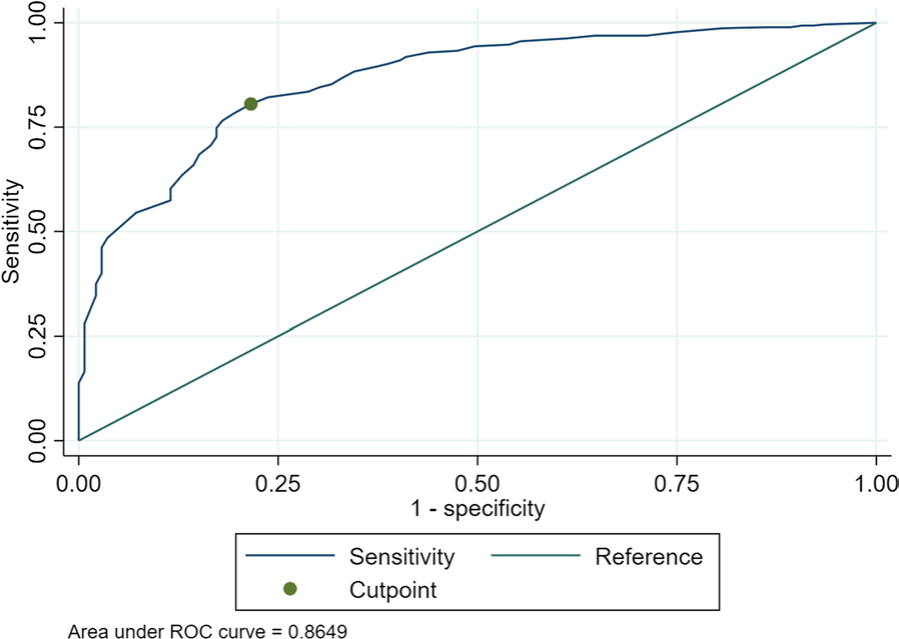
Receiver operating characteristic (ROC) curve for DAP score at cut-point < 2

Over the 12month study 14.0% (139/994) of women enrolled in the study at baseline were known to experience at least one pregnancy. The Youden recommended cut-point was 1.96 (rounded here to < 2), at which point the DAP had a sensitivity of 0.78 and specificity of 0.81. The AUROC was excellent (0.87) (Fig. 1), suggesting that DAP score is a good discriminator of whether someone will become pregnant in the next 12 months. The sensitivity tells us that 78% of all the women who will become pregnant over the next 12 months would be detected by using the DAP with a cut-point of < 2. In this sample the cumulative incidence of pregnancy over 12 months was 16% (95%CI 13%, 18.3%) making the PPV 43% and the NPV 95% at this cut-point. The PPV shows us that, in this sample, 43% of people with score of < 2 will become pregnant within 12 months.

Depending on the population and the purpose of the question the cut-point could be adapted to suit the purpose, as shown in Table 1. For example, if the identification of a preconception cohort was the goal a lower cut-point, such as < 0.5, yields a PPV of 73%, i.e., 73% of people with score of < 0.5 will become pregnant within 12 months. Conversely, using a cut-point of < 3, < 1% of people scoring over 3 will become pregnant within 12 months.

**Table 1:** Sensitivity, specificity, AuROC, positive and negative predictive values at a range of DAP cut-points

Cut-point	Sensitivity		Specificity		AuROC		PPV		NPV	
<0.5										
Estimate	17.3%		98.8%		0.58		72.7%		86.5%	
95% CI	11.4%	24.6%	97.7%	99.4%	0.55	0.61	54.5%	86.7%	84.0%	88.7%
<0.1										
Estimate	50.4%		94.4%		0.72		62.5%		91.1%	
95% CI	41.8%	58.9%	92.5%	95.9%	0.68	0.77	52.9%	71.5%	88.8%	93.0%
<0.2										
Estimate	78.4%		80.5%		0.80		42.9%		95.2%	
95% CI	70.6%	84.9%	77.5%	83.3%	0.76	0.83	36.7%	49.2%	93.3%	96.8%
<3										
Estimate	97.1%		46.2%		0.72		25.2%		98.9%	
95% CI	92.8%	99.2%	42.5%	49.8%	0.69	0.74	21.6%	29.1%	97.1%	99.7%

*AuROC* area under the area the receiver operator curve, *PPV* positive predictive value, *NPV* negative predictive value

### Predictive ability of the DAP

#### Univariate analysis

As previously reported [2], univariate analysis showed that for every one-point increase in DAP score the odds of pregnancy within 12 months decreased by 78% (OR 0.22, 95%CI 0.17, 0.28). Women with a DAP score of zero had a predicted 79.4% chance of pregnancy in the next 12 months, while 0.89% of those with a DAP score of four experienced pregnancy.

Preferences regarding future pregnancy varied by all five socio-demographic factors, as shown in Table 2. Desire to avoid pregnancy was highest in; the 15–19 age group; those not in a relationship; those with three or more children; women in Black, Asian, Mixed and Other ethnic groups; women whose highest completed level education was secondary (high) school (usually school up to age 18).

**Table 2:** Distribution of DAP score by socio-demographic variables

	N	Median	Inter quartile range		p value
Age group					< 0.001
15–19	139	3.50	3.00	3.86	
20–24	143	3.14	2.36	3.64	
25–29	139	2.29	1.57	2.93	
30–34	224	2.14	1.11	2.86	
35–39	209	2.57	1.57	3.21	
≥ 40	101	2.79	2.14	3.36	
Missing	39	2.71	2.14	3.29	
Relationship status					
Married/civil partnership	479	2.36	1.36	3.00	< 0.001
In a relationship	338	2.82	2.07	3.57	
Not in a relationship	152	3.36	2.86	3.79	
Missing	25	2.86	2.29	3.29	
Number of children in the household					
0	430	2.07	3.00	3.71	< 0.001
1	208	0.86	1.89	2.61	
2	233	2.07	2.79	3.29	
>/= 3	78	2.29	2.86	3.43	
Missing	45	2.29	2.93	3.29	
Grouped ethnicity					
White	834	1.79	2.64	3.36	0.042
BAME	128	2.11	2.82	3.64	
Missing	32	2.29	2.89	3.50	
Highest completed level of education					
School*	247	2.57	3.29	3.79	< 0.001
Undergraduate	389	1.57	2.50	3.14	
Postgraduate or professional qualifications	309	1.71	2.50	3.14	
Missing	31	2.14	2.93	3.64	

*includes those currently still at school taking GCSEs (n = 17) and one person with no qualifications

Likelihood of pregnancy in the next 12 months also varied by baseline measures of age, relationship status, number of children in the household and education level, but not by ethnicity. Pregnancy in the next 12 months was most likely to occur in the 30–34 age group compared to women aged 15–19 (OR 12.9 95%CI 3.9, 42.2), in women in a marriage or civil partnership compared to those who were not in a relationship (OR 9.55 95%CI 3.44, 26.5), women with one child in the household compared to women with none (OR 5.07 95%CI 3.22, 7.97) and women with completed undergraduate level educational attainment compared to those whose highest level of education was school (OR 4.61 95%CI 2.44, 8.71).

#### Multivariable analysis

In the development of the multivariable model, relationship status (p = 0.50), ethnicity (p = 0.43) and education level (p = 0.20) were not significantly associated with pregnancy when all factors were included. Only age and number of children remained in the final multivariable model. The relationship between DAP score and pregnancy was unchanged in the multivariable model and was the strongest predictor, as shown in Table 3.

**Table 3:** Multivariable logistic regression of the odds of pregnancy within 12 months

		N	Relationship with pregnancy between baseline and 12 months							
			Unadjusted estimates				Adjusted estimates			
			OR	95%CI		p value	OR	95%CI		p value
DAP score	0–4	884	0.22	0.17	0.28		0.22	0.17	0.29	< 0.001
Age group	15–19	139	Reference			< 0.001	Reference			0.025
	20–24	143	2.56	0.68	9.72		0.69	0.15	3.12	
	25–29	139	10.82	3.20	36.57		1.78	0.46	6.88	
	30–34	224	12.87	3.92	42.23		0.90	0.22	3.66	
	35–39	209	5.64	1.67	19.07		0.49	0.11	2.12	
	40 +	101	3.62	0.93	14.10		0.47	0.09	2.47	
Number of children	0	430	Reference			< 0.001	Reference			0.004
	1	208	5.07	3.22	7.97		3.30	1.74	6.26	
	2	233	1.24	0.72	2.15		2.56	1.16	5.65	
	3 +	78	0.88	0.36	2.17		2.40	0.78	7.41	
Relationship status	Not in a relationship	152	Reference			< 0.001	Not retained in final model			
	In a relationship	338	3.39	1.17	9.84					
	Married	479	9.55	3.44	26.47					
Ethnicity	White	834	Reference			0.31				
	Black, Asian, Mixed and Other ethnicities	128	0.74	0.41	1.34					
Highest education level	School	265	Reference			< 0.001				
	Undergraduate	389	4.61	2.44	8.71					
	Postgraduate	309	3.79	1.97	7.31					

*OR* odds ratio, *95%CI* 95% confidence interval

**Fig. 2:**
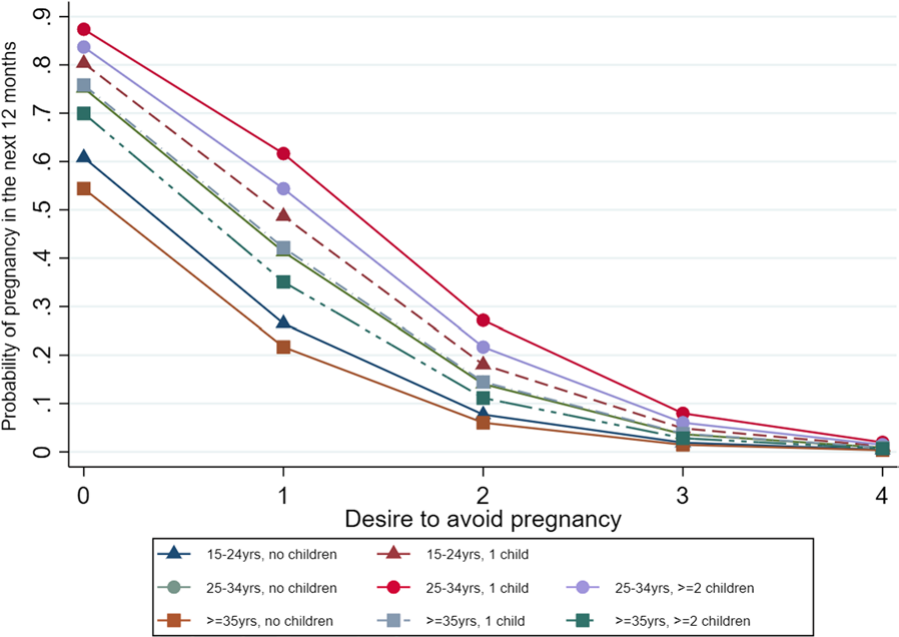
Predicted probability of pregnancy within 12 months based on DAP score taking into account age group and number of children in the household

The predicted probabilities of pregnancy according to selected age groups and number of children estimated by the multivariate model can be seen in Fig. 2. The probability of pregnancy was highest (87.4%) in women in aged 25–34 who already had one child in the household and scored zero on the DAP score at baseline (indicating that they desired a pregnancy). Regardless of age or number of children, women with a DAP score of four at baseline were very unlikely (< 2%) to have a pregnancy within the next year. Women who were aged 35 and over with no children but who scored zero on the DAP at baseline had a 54.4% chance of pregnancy.

### Time frames

The association between baseline DAP score and pregnancy did not differ across time points as shown in Table 4*.*

**Table 4:** Odds of pregnancy (OR) at each follow-up by baseline DAP score with 95% confidence interval (95% CI)

Relationship between DAP score and pregnancy at:			
	OR	95%CI	
3 months	0.24	0.17	0.34
6 months	0.19	0.14	0.26
9 months	0.18	0.13	0.24
12 months	0.22	0.17	0.28

*95%CI* 95% confidence interval

## Discussion

This is the first study to examine the potential utility of the DAP scale as a tool to predict future pregnancy. Based on this analysis, the DAP could be used, with a cut-point selected based on the purpose, to identify who is likely to become pregnant over the next 12 months and who is not.

While pregnancy desire and occurrence are associated with a range of socio-demographic factors, both in our data and the wider literature [5–7], the DAP score is the most strongly associated factor, based on the size of the odds ratio and level of statistical significance, even when other factors are taken into account. There were differences in the probability of pregnancy in those with a DAP score of zero based on age and number of children, in keeping with the literature [5, 6, 12]. Women in their late 20s and early 30s who already had one child were the most likely to become pregnant in the time period studied. This is potentially due to them being in the phase of their life where they are building their family and norm of having two children. There was almost no difference in the probability of pregnancy, regardless of age or number of children, in those with the highest desire to avoid pregnancy. This demonstrates the gap between wanting something and the ability to make it happen which, in the case of pregnancy, is only within a person’s control to some extent. Pregnancy may be further affected by external factors such as fecund ability and the supportability of pregnancy, defined by MacLeod as ‘as the capacity of a woman to carry a pregnancy in such a way that she experiences positive health and welfare’ [13]. However, the overall predicted probability of pregnancy was remarkably congruent with evidence that approximately 80% of couples who desire pregnancy will conceive within 12 months [14–16]. There have been few attempts to develop predictive models for pregnancy, and many of those have focused on sub-fertile couples [17–19]. AUROCs for pregnancy prediction models in reproductive health have generally been low, ranging from 0.56 to 0.67, which may in part reflect the challenges of predicting pregnancy in a heterogeneous sub-fertile population [20]. One study, of women who were trying to conceive and who enrolled in a preconception health study, used machine learning to develop a prediction model from a pool of 163 potential predictors encompassing socio-demographics, diet and lifestyle, medical history and some partner characteristics [12]. The authors developed multiple models, considering different time frames and populations, with AUROCs between 0.65 and 0.71. In comparison to these other models, the 14-item DAP’s AUROC of 0.87 is very high. This is especially noteworthy as participants were not all trying to conceive and therefore may be more representative of the general population and of how the DAP may perform in practice.

The cut-point, and therefore sensitivity and specificity, could be varied by setting and purpose for administering the DAP. For example, in the context of a screening tool in primary care to identify who would benefit from preconception advice a lower cut-point could be used as it would be important to reduce false positives (i.e., women predicted to get pregnant who will not) as this could overload services and be unsustainable and unacceptable. Alternatively, for researchers planning a trial of a teratogenic agent and wishing to reduce attrition, one might select a high cut-point to ensure the lowest proportion of participants experience pregnancy.

### Strengths and limitations

The analysis has been conducted on a large cohort with little loss to follow-up. While a non-probability sample, comparison of the socio-demographics of the cohort indicate that is broadly representative of the UK population [2] suggesting generalisability of our findings to a primary care population. Importantly the cohort was neither sub-fertile nor self-identified as preconception, a distinction from previous pregnancy prediction model cohorts. We have demonstrated the high discrimination of the full DAP scale in excess of previously developed pregnancy prediction models.

While we included a range of sociodemographic factors known to be associated with pregnancy preferences in our multivariable model, we did not include all factors associated with fertility, such as body mass index or smoking [21] as we did not have data on these. Inclusion of these variables may have strengthened the model. Assessing the DAP scale’s performance in other languages, cultural settings and exploring pregnancy preferences by sexuality and in people of all genders will be important next steps.

The DAP scale could be useful clinically in a range of self-completion or digital formats to identify who is likely to become pregnant and who is not. Further work comparing individual items and combinations of items from the DAP with other questions about pregnancy preferences suggests that a shorter clinical screening tool for use in a face-to-face encounter, based on the DAP and retaining good sensitively and specificity, is possible [22] and implementation is a critical next step.

## Conclusion

This study shows the excellent predictive ability of the DAP, which was the strongest predictor of pregnancy even when other socio-demographic factors were taken into account. The estimates of the predicted probability of pregnancy using the DAP score are stable, suggesting it may not need to be asked more than once a year. At a cut-point of < 2 sensitivity and specificity are optimised at 78% and 81% respectively, however the information on the sensitivity and specificity of the DAP at a range of cut-points will support academics and clinicians to adapt the choice of cut-point according to their aim and needs.

### Supplementary Information

**Additional file 1.** Desire to Avoid Pregnancy Scale – wording of UK version.

**Additional file 2.** Comparison of women in the 12m follow up with those who were lost to follow up.

## Data Availability

The dataset is available in the UCL Research Data Repository.

## References

[1] Rocca CH, Ralph LJ, Wilson M, Gould H, Foster DG (2019). Psychometric evaluation of an instrument to measure prospective pregnancy preferences: the desire to avoid pregnancy scale. Med Care.

[2] Hall J, Barrett G, Rocca C (2022). Evaluation of the Desire to Avoid Pregnancy Scale in the UK: a psychometric analysis including predictive validity. BMJ Open.

[3] Samari G, Foster DG, Ralph LJ, Rocca CH (2020). Pregnancy preferences and contraceptive use among US women. Contraception.

[4] Rocca CH, Smith MG, Hale NL, Khoury AJ (2022). Ranges of pregnancy preferences and contraceptive use: results from a population-based survey in the southeast United States. Perspect Sex Reprod Health.

[5] Hill B, Ling M, Mishra G, Moran LJ, Teede HJ, Bruce L (2019). Lifestyle and psychological factors associated with pregnancy intentions: findings from a longitudinal cohort study of australian women. Int J Environ Res Public Health.

[6] Lang AY, Harrison CL, Barrett G, Hall JA, Moran LJ, Boyle JA (2021). Opportunities for enhancing pregnancy planning and preconception health behaviours of Australian women. Women Birth.

[7] Geist C, Everett BG, Simmons RG, Sanders JN, Gawron LM, Myers K (2021). Changing lives, dynamic plans: prospective assessment of 12-month changes in pregnancy timing intentions and personal circumstances using data from HER Salt Lake. PLoS ONE.

[8] Harris PA, Taylor R, Minor BL, Elliott V, Fernandez M, O'Neal L (2019). The REDCap consortium: building an international community of software platform partners. J Biomed Inform.

[9] Harris PA, Taylor R, Thielke R, Payne J, Gonzalez N, Conde JG (2009). Research electronic data capture (REDCap)–a metadata-driven methodology and workflow process for providing translational research informatics support. J Biomed Inform.

[10] Youden WJ (1950). Index for rating diagnostic tests. Cancer.

[11] Hosmer DW, Lemeshow S (2000). Applied Logistic Regression.

[12] Yland JJ, Wang T, Zad Z, Willis SK, Wang TR, Wesselink AK (2022). Predictive models of pregnancy based on data from a preconception cohort study. Hum Reprod.

[13] Macleod CI (2016). Public reproductive health and ‘unintended’ pregnancies: introducing the construct ‘supportability’. J Public Health (Oxf).

[14] Gnoth C, Godehardt D, Godehardt E, Frank-Herrmann P, Freundl G (2003). Time to pregnancy: results of the German prospective study and impact on the management of infertility. Hum Reprod.

[15] Potter RG, Parker MP (1964). Predicting the time required to conceive. Popul Stud.

[16] Zinaman MJ, Clegg ED, Brown CC, O'Connor J, Selevan SG (1996). Estimates of human fertility and pregnancy loss. Fertil Steril.

[17] Hunault CC, Habbema JD, Eijkemans MJ, Collins JA, Evers JL, te Velde ER (2004). Two new prediction rules for spontaneous pregnancy leading to live birth among subfertile couples, based on the synthesis of three previous models. Hum Reprod.

[18] Hunault CC, Laven JS, van Rooij IA, Eijkemans MJ, te Velde ER, Habbema JD (2005). Prospective validation of two models predicting pregnancy leading to live birth among untreated subfertile couples. Hum Reprod.

[19] van der Steeg JW, Steures P, Eijkemans MJ, Habbema JD, Hompes PG, Broekmans FJ (2007). Pregnancy is predictable: a large-scale prospective external validation of the prediction of spontaneous pregnancy in subfertile couples. Hum Reprod.

[20] Coppus SF, van der Veen F, Opmeer BC, Mol BW, Bossuyt PM (2009). Evaluating prediction models in reproductive medicine. Hum Reprod.

[21] Oostingh EC, Hall J, Koster MPH, Grace B, Jauniaux E, Steegers-Theunissen RPM (2019). The impact of maternal lifestyle factors on periconception outcomes: a systematic review of observational studies. Reprod Biomed Online.

[22] Hall JA, Barrett G, Stephenson JM, Edelman NL, Rocca C (2023). Desire to Avoid Pregnancy scale: clinical considerations and comparison with other questions about pregnancy preferences. BMJ Sex Reprod Health..

