# Supplementary material for: Predictive ability of the Desire to Avoid Pregnancy scale
Source: Reprod Health. 2023 Sep 25;20:144. doi: 10.1186/s12978-023-01687-9 (PMC10521409; doi:10.1186/s12978-023-01687-9)
Supplement: Supplementary file 1 — Additional file 1. Desire to Avoid Pregnancy Scale – wording of UK version. [file 12978_2023_1687_MOESM1_ESM.docx]

**Desire to Avoid Pregnancy Scale – wording of UK version**

We would like to ask you about your current thoughts and feelings about the idea of becoming pregnant in the next three months and having a baby in the next year.

We know that women have very different thoughts and feelings about pregnancy, and having a baby, that can change over time.

Please remember that there are no right or wrong answers. For each question, choose one answer that seems right at this point in your life.

The following questions ask about your thoughts and feelings about the idea of becoming PREGNANT IN THE NEXT THREE MONTHS.

Even if you do not think you can become pregnant for partner or physical reasons, please imagine how you feel about BECOMING PREGNANT in the next 3 months.

(5 point Likert scale from Strongly agree to Strongly disagree)

I wouldn't mind if I became pregnant in the next 3 months. (Item 1)

It would be a good thing for me if I became pregnant in the next 3 months. (Item 2)

Thinking about becoming pregnant in the next 3 months makes me feel unhappy. (Item 3)

Thinking about becoming pregnant in the next 3 months makes me feel excited. (Item 4)

The next questions ask you to think about your main partner. By main partner, we mean the romantic partner that is the most serious to you.

If you don't have a romantic partner, please think about the last person with whom you were physically intimate (anything from kissing and cuddling to sex) or think of a person you know who you would consider being physically intimate with.

Becoming pregnant in the next 3 months would bring me closer to my main partner. (Item 5)

The following questions ask about your thoughts and feelings about the idea of having a BABY IN THE NEXT YEAR.

Even if you do not think you can have a baby for partner or physical reasons, please imagine how you feel about HAVING A BABY in the next year.

(5 point Likert scale from Strongly agree to Strongly disagree)

I want to have a baby within the next year. (Item 6)

If I had a baby in the next year, it would be bad for my life. (Item 7)

It would be a positive addition to my life to have a baby in the next year. (Item 8)

It would be the end of the world for me to have a baby in the next year. (Item 9)

Thinking about having a baby within the next year makes me smile. (Item 10)

Thinking about having a baby within the next year makes me feel stressed out. (Item 11)

I would feel a loss of freedom if I had a baby in the next year. (Item 12)

If I had a baby in the next year, it would be hard for me to manage raising the child. (Item 13)

I would worry that having a baby in the next year would make it harder for me to achieve other things in my life. (Item 14)
