# Supplementary material for: Predictive ability of the Desire to Avoid Pregnancy scale
Source: Reprod Health. 2023 Sep 25;20:144. doi: 10.1186/s12978-023-01687-9 (PMC10521409; doi:10.1186/s12978-023-01687-9)
Supplement: Supplementary file 2 — Additional file 2. Comparison of women in the 12m follow up with those who were lost to follow up. [file 12978_2023_1687_MOESM2_ESM.docx]

# Supplementary information – S2

## Comparison of women in the 12m follow up with those who were lost to follow up

## Cohort

89.8% (807/899) of those eligible for the 12m follow up completed it

This is a comparison of 12m sample with those not in the 12m sample but who did not have an ongoing pregnancy

## Age

Women’s age in years was compared with a paired t-test between those who were eligible and did or did not complete the 12m follow up. While women who did not complete 12 m follow up were on average slightly younger (n=92, mean 28.2 years, 95%CI 26.3, 30.1, for non-completers and n=807, mean 29.9 years, 95%CI 29.3, 30.5 for completers) this was not statistically significant p=0.057.

## Ethnicity

At baseline 86.5% of the cohort was white and 13.7% were from BAME groups; at 12months this was 87.3% and 12.7% which was not significant on chi-squared test p=0.739

## Relationship

Chi-squared test showed no significant difference between the relationship status of those who did and did not complete 12month follow up (p=0.269). At baseline 48.4% were married compared to 47.9% at 12 months.

## Number of children in the household

There was no significant difference in the number of children in the household of those who did and did not complete 12 month follow up on chi-squared testing p=0.519

## DAP score

Baseline DAP score between completers and non-completers at 12months was compared using the Kruskall-Wallis test given the non-normal distribution of the DAP and this was not significant p=0.4288. The baseline score in those who did complete 12m follow up was 2.67 compared to 2.60 in completers.
